# Effect of High-Dose Marine Omega-3 Fatty Acids on Atherosclerosis: A Systematic Review and Meta-Analysis of Randomized Clinical Trials

**DOI:** 10.3390/nu11112599

**Published:** 2019-10-30

**Authors:** Akira Sekikawa, Chendi Cui, Daisuke Sugiyama, Anthony Fabio, William S. Harris, Xiao Zhang

**Affiliations:** 1 Department of Epidemiology, Graduate School of Public Health, University of Pittsburgh, Pittsburgh, PA 15213, USA; chc217@pitt.edu (C.C.); dsugiyama@keio.jp (D.S.); anthony.fabio@pitt.edu (A.F.); XIZ186@pitt.edu (X.Z.); 2 Faculty of Nursing and Medical Care, Keio University, 4411 Endo, Fujisawa, 252-0883 Kanagawa, Japan; 3 OmegaQuant Analytics, LLC and Sanford School of Medicine, University of South Dakota, Sioux Falls, SD 57106, USA; Bill@omegaquant.com

**Keywords:** Marine omega-3 fatty acids, atherosclerosis, high-dose, meta-analysis, systematic review, randomized clinical trial, mechanism

## Abstract

A recent randomized controlled trial (RCT), the Reduction of Cardiovascular Events with Icosapent Ethyl-Intervention Trial (REDUCE-IT), reported that high-dose marine omega-3 fatty acids (OM3) significantly reduce cardiovascular disease (CVD) outcomes, yet the mechanisms responsible for this benefit remain unknown. To test the hypothesis that high-dose OM3 is anti-atherosclerotic, we performed a systematic review and meta-analysis of RCT of high-dose OM3 on atherosclerosis. The protocol of this systematic review was registered with PROSPERO (CRD42019125566). PubMed, Embase, Cochran Central Register for Controlled Trials, and Clinicaltrials.gov databases were searched using the following criteria: adult participants, high-dose OM3 (defined as ≥3.0 g/day, or in Japan 1.8 g/day and purity ≥90%) as the intervention, changes in atherosclerosis as the outcome, and RCTs with an intervention duration of ≥6 months. A random-effects meta-analysis was used to pool estimates across studies. Among the 598 articles retrieved, six articles met our criteria. Four RCTs evaluated atherosclerosis in the coronary and two in the carotid arteries. High-dose OM3 significantly slowed the progression of atherosclerosis (standardized mean difference −1.97, 95% confidence interval −3.01, −0.94, *p* < 0.001). The results indicate that anti-atherosclerotic effect of high-dose OM3 is one potential mechanism in reducing CVD outcomes demonstrated in the REDUCE-IT trial.

## 1. Introduction 

A recent randomized controlled trial (RCT) of 4 g/day of marine omega3 fatty acids (OM3), specifically 4 g/day of icosapent ethyl (3.84 g/day of eicosapentaenoic acid (EPA)) on cardiovascular disease (CVD), the Reduction of Cardiovascular Events with Icosapent Ethyl-Intervention Trial (REDUCE-IT), showed a significant 25% relative risk reduction in CVD outcomes compared to the control group. The REDUCE-IT trial was conducted among 8,179 statin-treated patients with CVD or diabetes and with high triglycerides (TG) and low low-density lipoprotein cholesterol (LDL-C) [1]. This result is in striking contrast to the results of recent RCTs of low-dose (≤1 g/day) OM3 that showed no significant effect of OM3 on CVD outcomes in a variety of groups: subjects with CVD (Alpha-Omega [2] and Supplementation en Folates et Omeag-3 [3]), without CVD (Vitamin D and Omega-3 Trial [4]), with multiple CVD risk factors (Risk and Prevention Study [5]), diabetes (A Study of Cardiovascular Events iN Diabetes [6]), or glucose intolerance (the Outcome Reduction with an Initial Glargine Intervention [7]). The rationale for the dosage of REDUCE-IT (4.0 g/day of OM3) was to mimic the blood levels of OM3 observed in the intervention group in the Japan eicosapentaenoic acid (EPA) Lipid Intervention Study (JELIS), [1,8] an RCT in Japan of EPA alone that showed a significant 19% relative risk reduction in CVD outcomes compared to the control group in 18,645 statin-treated subjects with and without CVD [9]. JELIS used 1.8 g/day of OM3, equivalent to 3.6 g/day of OM3 in the US, due to the very high dietary intake of OM3 in Japan [8]. These results indicate that high- but not low-dose OM3 reduces CVD outcomes. 

Neither REDUCE-IT [1] nor JELIS [9] has directly tested the mechanism of high-dose OM3. OM3 has varying effects on traditional risk factors (blood pressure, high-density lipoprotein cholesterol (HDL-C), TG, etc.). These effects are modest at best, even with a high-dose OM3 (4 g/day) [10,11] except for lowering TG among patients with high TG [12]. However, the significant reduction in CVD outcomes in these two trials does not depend on baseline TG levels [1,9], suggest that lowering TG is unlikely to be a major mechanism of high-dose OM3 in reducing CVD outcomes. In fact, REDUCE-IT investigators recently reported that the cardiovascular benefits were primarily tied to non-TG-related effects [13]. OM3 has other effects on the cardiovascular system, including anti-atherosclerotic, anti-inflammatory, anti-thrombotic, and anti-arrhythmic properties [12,14,15]. Investigators of the REDUCE-IT trial discussed that the benefit of high-dose OM3 seen in this trial was due in part to the anti-atherosclerotic and anti-inflammatory properties [1]. They also speculated that the benefit was not due to anti-thrombotic or anti-arrhythmic properties [1]. This speculation is in accordance with recent reviews that anti-thrombotic or anti-arrhythmic properties are unlikely pathways of high-dose OM3 (4 g/day) for reducing CVD outcomes [12,16].

A recent RCT in the US of 3.4 g/day of OM3 in patients with coronary heart disease (CHD) reported slower progression of coronary plaques [17]. Moreover, three RCTs of 1.8 g/day of OM3 in Japan (equivalent to 3.6 g/day of OM3 in the US) showed significantly slower progression of coronary atherosclerosis in patients with CHD in the intervention group as compared to the control group [18,19,20]. However, no systematic review and meta-analysis of RCTs has been conducted on the effect of high-dose OM3 on atherosclerosis. In this systematic review and meta-analysis, we hypothesized that high-dose OM3 significantly slows the progression of atherosclerosis. 

## 2. Materials and Methods

This article has been reported in accordance with the Preferred Reporting Items for Systematic Reviews and Meta-Analyses [21]. The protocol was registered with PROSPERO (CRD42019125566). A systematic search of PubMed, Embase, Cochran Central Register of Controlled Trials and clinicaltrials.gov was conducted from the earliest publication date through March 1, 2019. The reference lists of included studies in the search were also screened for additional studies. The search strategies are available in Tables S1 and S2. After removal of duplicates, the titles and abstracts were retrieved by five authors (AS, CC, DS, XZ, WH) to select relevant studies. Then, full-texts were independently retrieved by pairs of authors (AS, CC, DS, XZ) and the final list of studies was determined by discussion, including minor differences being resolved with another author (AF). 

The studies included were RCTs (1) conducted among adults (≥18 years) without hemodialysis, (2) using high-dose OM3 supplements (defined as ≥3 g/day of OM3 or ≥1.8 g/day of OM3 in Japan) with purity of OM3 ≥90% as the intervention, (3) using atherosclerosis as the primary outcome, (4) reporting percent or absolute change of atherosclerosis, (5) with the intervention period ≥6 months, and (6) with articles published and available in full-text English language.

The data extracted from each selected RCT included characteristics and demographics (first author, publication year, study location, study design, etc.), baseline participant’ characteristics (total sample size, age, health condition, etc.), dose and purity of OM3, duration of intervention, methods to assess atherosclerosis, baseline and post-pre-intervention change in atherosclerosis, and net difference in change of atherosclerosis. Only the primary outcome was extracted when multiple endpoints were reported.

Risk of bias assessment was evaluated independently by four investigators (AS, CC, DS, and XZ) using the Cochrane Collaboration’s tool for assessing risk of bias across seven domains (random sequence generation, allocation concealment, blinding of participants and personnel, blinding of outcome assessment, incomplete outcome data, selective outcome reporting, and other source of bias) [22]. The judgment of high, low, or unclear risk of bias was assigned for each item. When more than four domains were regarded as high quality, the study was considered to be at low risk.

### Statistical analysis

Mean change from baseline of atherosclerotic measurements was calculated for the treatment and control groups, and the standardized mean difference (SMD) was computed as the measure of effect [23]. The pooled SMD across studies (with the 95% confidence intervals (CIs)) was calculated based on a random effect model:

(1) $$SMD=\;\frac{\bar{X_{treatment}}-\;\bar{X_{control}}}{S_{pooled}},$$

where

(2) $$S_{pooled}=\sqrt{\frac{\left(n_{treatment}-1\right)*S_{treatment}^{2}+\left(n_{control}-1\right)*S_{control}^{2}}{n_{treatment}+n_{control}\;}}.$$

For studies where the mean change and standard deviation (SD) was not available, SMD was estimated using the median change, interquartile range (IQR), and sample size [24,25]. For results in which positive values represented improvement (e.g., fibrous cap thickness), the estimate was multiplied by −1 to make the direction of the result consistent with the results of other RCTs. Heterogeneity of the studies was assessed with the I^2^ statistic to describe the percentage of variation across each study that may be due to heterogeneity rather than chance. In addition, a funnel plot was generated to identify potential publication bias or systematic heterogeneity. Because we detected significant heterogeneity, sensitivity analyses were performed by removing the most influential study followed by removing the next influential study. 

To assess whether the effect of OM3 on atherosclerosis differed by study characteristics, a subgroup analysis was conducted by location (Japan vs. other countries), site of atherosclerosis (coronary vs. carotid arteries), placebo-controlled (yes vs. no), CHD (yes vs. no), statin use (yes vs. no), source of OM3 (EPA only vs. EPA + docosahexaenoic acid (DHA)), and risk of bias (high vs. low). All analyses were conducted with Cochrane Review Manager 5.3 [23]. The level of significance was set at *p* < 0.05. Subgroup analyses were assessed with Bonferroni correction for multiple comparisons. 

## 3. Results

**Figure 1:**
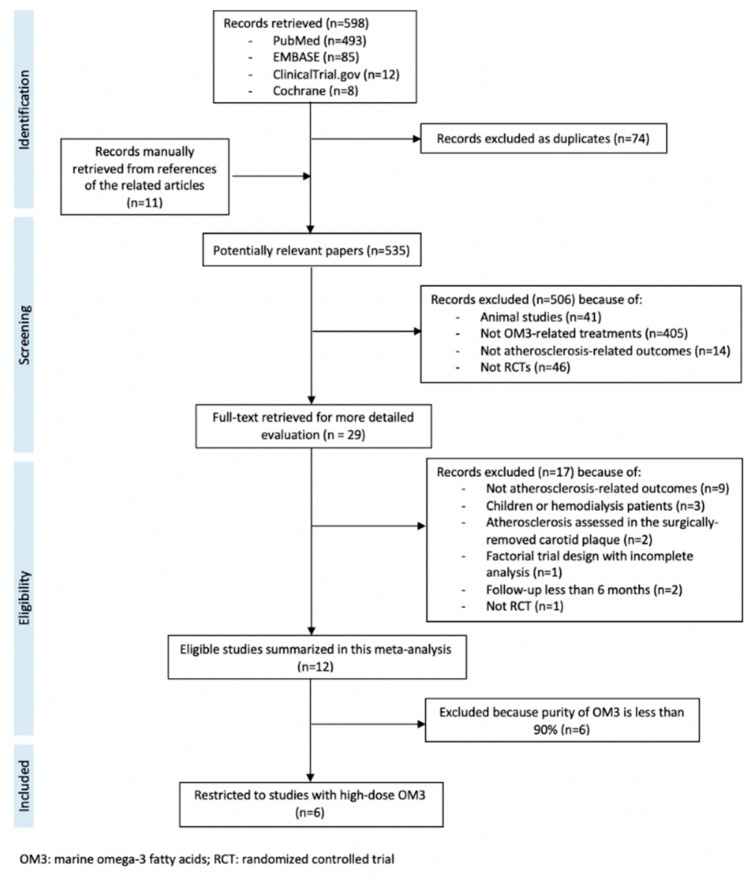
PRISMA flow chart of meta-analysis.

Of the 598 articles retrieved, 12 studies were identified that met our review criteria. Among the 12 RCTs, six were RCTs of high-dose OM3 with purity of OM3 ≥90% and were included in the analysis (Figure 1) [17,18,19,20,26,27]. These trials were published as early as 2006 but most were published in 2016 and 2017 (Table 1). These RCTs were conducted in Japan (*n* = 4), the US (*n* = 1) and the UK (*n* = 1). The participants (a total of 693, 71% male) were patients with CHD (*n* = 4), [17,18,19,20] type 2 diabetes (*n* = 1) [26] or nonalcoholic fatty liver disease (*n* = 1) [27]. Participants in four RCTs were treated with a statin. Only one study was placebo-controlled and the other five were open-label trials with no placebo. The dose of OM3 ranged from 1.8 to 3.36 g/day. All the four RCTs conducted in Japan used highly-purified EPA [18,19,20,26] whereas the other three studies used a combination of EPA and DHA [17,27]. The average follow-up duration ranged from 6 to 30 months (Table 1).

The four RCTs that recruited patients with CHD evaluated atherosclerosis in the coronary artery using various methods (coronary computed tomography angiography [17], intravascular ultrasound [19,20], and optical, coherence tomography [18]) (Table 2). The primary outcome differed for each RCT (% change in non-calcified plaque volume [17], change in lipid plaque volume [19], change in fibrous-cap thickness [18], and change in normalized total atheroma volume [20]). Two RCTs that recruited non-alcoholic fatty acid disease [27] or type 2 diabetes [26] evaluated atherosclerosis in the carotid artery with B-mode ultrasound. One RCT used change in mean intima-media thickness (IMT) [27] whereas another study used change in max IMT of the carotid artery [26] as the primary outcome. These four RCTs documented that the OM3 significantly slowed the progression of atherosclerosis as compared to the control group [18,19,20,26] (Table 2).

Three out of the six trials were considered to have high risk of bias (score <5) (Table 3). They were open label trials without placebo. Two of the three trials did not provide enough information on random sequence generation, allocation concealment, and selective outcome reporting.

**Figure 2:**
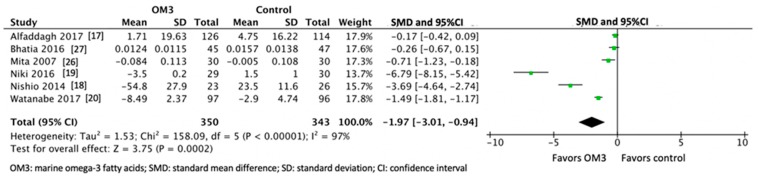
Effect of high-dose marine omega-3 fatty acids on atherosclerosis.

**Figure 3:**
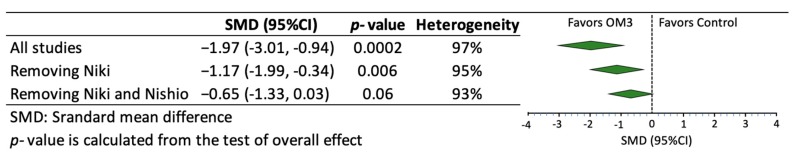
Sensitivity analysis after excluding the most and second most influential studies.

Overall, OM3 significantly slowed the progression of atherosclerosis (SMD −1.97, 95% confident interval (95%CI) −3.01, −0.94: *p* < 0.001). A significant heterogeneity was observed across these studies (I^2^ = 97%, *p* < 0.001) (Figure 2). Our sensitivity analysis showed that after excluding the most influential study, OM3 significantly slowed the progression (SMD −1.17 95% CI −1.99, −0.34: *p* = 0.006.) After further excluding the next influential study, the effect was attenuated and became non-significant (SMD −0.65 95% CI −1.33, 0.03: *p* = 0.06) (Figure 3).

**Figure 4:**
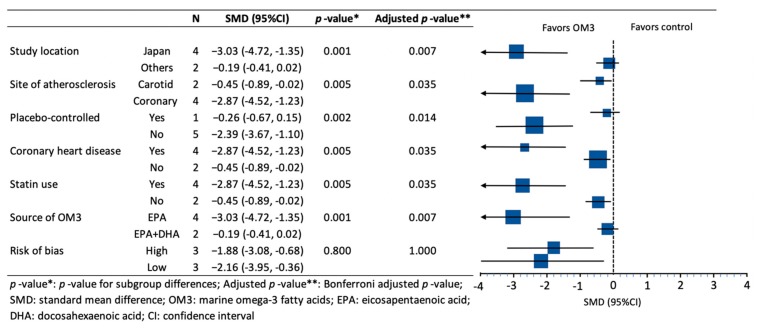
Subgroup analysis of the meta-analysis.

In subgroup analysis, the effect of OM3 on atherosclerosis differed significantly by study location (Japan vs. other countries), site of atherosclerosis (coronary vs. carotid arteries), use of placebo (placebo-controlled trials vs. trials without placebo), presence of CHD (yes vs. no), statin use (yes vs. no), and source of OM3 (EPA vs. a combination of EPA and DHA). However, the subgroup analysis showed that the effect was not significantly different by risk of bias (high vs. low) (Figure 4). 

## 4. Discussion

This systematic review and meta-analysis of six RCTs involving 693 participants demonstrated that randomization to high-dose OM3 supplementation significantly slowed the progression of atherosclerosis as compared to a control group. Although a significant heterogeneity was observed across these six RCTs, after removing the most influential study, the effect of high-dose OM3 on atherosclerosis remained significant, which was attenuated and became non-significant (*p* = 0.06) after further excluding the second influential study. 

Studies in basic science have shown that OM3 is anti-atherosclerotic through various molecular mechanisms [28,29]. Preclinical studies document that OM3 slows the progression of atherosclerosis in mice, [30,31,32,33,34] swine [35], and monkey [36]. However, observational studies in humans have shown mixed results. Generally, studies in Western countries reported that dietary intake or blood levels of OM3 show no significant associations with atherosclerosis. The Atherosclerosis Risk in the Community Study [37] and Multi-Ethnic Study of Atherosclerosis (MESA) [38] in the US report that dietary intake or blood levels of OM3 have no significant association with carotid IMT. Likewise, the Rotterdam Study in the Netherlands [39] and MESA [38] report no significant association of dietary intake of OM3 with coronary artery calcification (CAC), a well-established biomarker of coronary atherosclerosis [40]. On the other hand, studies in Japan show that dietary intake or blood levels of OM3 have a significant inverse association with IMT [41] and CAC [42]. Notably, we have reported from our international population-based study in Japan and the US that blood levels of OM3 are >100% higher in Japanese and a significant inverse association of blood levels of OM3 with IMT is observed only in Japanese and not in Americans [43]. Collectively, these observations suggest that high but not low levels of OM3 are anti-atherosclerotic. 

We excluded RCTs of OM3 with purity of OM3 <90% according to a recent review paper on the differential effect of EPA and DHA on cardiometabolic factors [44]. In the process, we identified five RCTs that administered low-dose OM3 (dose <3 g/day) with purity of OM3 <90% [45,46,47,48,49] (Table S3). Characteristics of participants in these trials were similar to those in high-dose trials (patients with CHD or dyslipidemia). The duration of intervention was somewhat longer in these trials (12 to 50 months) compared to the high-dose trials (6 to 28 months). The dose of OM3 ranged from 0.84 to 2.52 g/day. Changes in atherosclerosis were assessed in the carotid artery using ultrasound (*n* = 3) and in the coronary artery by intra-vascular ultrasound (*n* = 1) and quantitative coronary angiography (*n* = 1) (Table S4). None of these studies showed any significant difference in change in atherosclerosis between the intervention and control groups (Table S5). Furthermore, synthesizing the data from these five studies showed no significant effect of low-dose OM3 (SMD 0.02 95% CI −0.08, 0.13, *p* = 0.64) (Figure S1). This observation further supports the notion that a high but not low dose of OM3 is anti-atherosclerotic in humans.

In our subgroup analysis, the effect of high-dose OM3 was significantly different in six of seven categories even after the Bonferroni correction (study location, site of atherosclerosis, use of placebo, statin use, presence of CHD, and source of OM3). The results were largely due to four RCTs conducted in Japan that administered 1.8 g/day of EPA without placebo and reported significantly slower progression of atherosclerosis [18,19,20,26] and three RCTs that evaluated coronary arteries of patients with CHD who were on statins [18,19,20]. Therefore, whether the effect of high-dose OM3 differs in relationship to these factors remain unknown. However, because both JELIS (1.8 g/day of EPA) in Japan [9] and REDUCE-IT (4.0 g/day of icosapent ethyl (3.84 g/day of EPA)) primarily in Western countries [1] showed significant relative risk reduction in CVD outcomes, it is unlikely that the effect is different by study location. 

A recent review on the interaction between statin and OM3 reports that statin and OM3 have both synergistic and antagonistic effects [50]. Both statin and OM3 have pleiotropic effects which overlap. Statins are standard therapy for both secondary and primary prevention of CVD and high-dose OM3 is considered to be an add-on therapy to statins based on the results of JELIS and REDUCE-IT (both of which show that high-dose OM3 is effective in reducing CVD outcome in statin-treated patients). Whether high-dose OM3 reduces CVD outcomes without statin-treatment remains to be answered. 

There is strong evidence that use of EPA alone without DHA significantly reduces CVD outcomes [1,9] and slows the progression of atherosclerosis [18,19,20,26]. The investigators of REDUCE-IT have stated that the results may not be extrapolated into a combination of EPA and DHA because DHA but not EPA raises LDL-C. In fact, two meta-analyses have reported that a combination of EPA and DHA raises LDL-C by 5 mg/dL [51,52]. However, the American Heart Association Science Advisory on OM3 for the management of hypertriglyceridemia recently reported that EPA or a combination of EPA and DHA does not increase LDL-C in individuals with hypertriglyceridemia (TG levels of 2.26–5.64 mmol/L) although a combination of EPA and DHA may increase LDL-C among individuals with very high TG (above 5.65 mmol/L) [53]. In this regard, the results of the Outcomes Study to Assess Statin Residual Risk Reduction with EpaNova in HiGh CV risk PatienTs with Hypertryglyceridemia (STRENGTH) are awaited, an ongoing trial of a combination of high-dose EPA and DHA on CVD outcomes in approximately 13,000 patients with hypertriglyceridemia [54]. 

Several RCTs have reported the effect of high-dose EPA or DHA on CVD risk factors including lipids and lipoproteins [55,56,57,58]. As compared to EPA, DHA significantly decreases TG, [57,58] increases LDL particle size, [55] LDL-C [57], and HDL-C [55,57] while the effect on apolipoprotein B is similar [57]. Interestingly, one of these RCTs showed that, as compared to EPA, DHA is more effective in modulating biomarkers of inflammation [57]. However, no RCTs including ongoing trials are testing the effect of DHA alone on vascular outcomes (e.g., atherosclerosis or CVD). 

Atherosclerosis is a chronic inflammatory disease [59]. Both innate and adaptive immunities are involved in the initiation and progression of atherosclerosis through plaque rupture [60]. OM3 has anti-inflammatory properties that have been applied to treating inflammatory diseases such as rheumatoid arthritis and inflammatory bowel disease [61]. Although reviewing the potential mechanisms linking OM3 and its anti-atherosclerotic properties through its anti-inflammatory effects is beyond the scope of the current paper, we describe several potential mechanisms. Interleukin-6 (IL-6) and C-reactive protein (CRP) are general biomarkers of inflammation in innate immunity and are independent predictors of future CVD events [62]. A recent systematic review and meta-analysis shows that administration of OM3 significantly reduces low-grade inflammation assessed by IL-6 and CRP in middle-age and older adults [63]. Evidence for a role of ceramides in the etiology of atherosclerosis and CVD is rapidly accumulating [64,65,66]. Untargeted metabolomics identified a significant association of ceramides with CVD [67,68]. Several observational studies reported a significant association of plasma concentrations of ceramides with CVD events independent of traditional risk factors [69,70,71]. Ceramides promote LDL infiltration of the endothelial cells and are upregulated in response to inflammatory cytokines, e.g., IL-6 [65]. Preclinical studies show that OM3 reduces plasma ceramides [72,73,74]. Predominant T cells found in atherosclerotic plaque are type 1 helper T cells (Th1) [75], which are pro-inflammatory and a biomarker of adaptive immunity [60]. It was reported in MESA among 917 men and women in the US general population that Th1 bias had significant positive associations with both CAC and IMT [76]. Preclinical studies show that administration of OM3 reduces the differentiation of native T cells to Th1 [77]. 

OM3 is incorporated into the cell membrane, modulates local signaling, and exerts anti-inflammatory effects by competing with omega-6 fatty acid [15]. OM3 can be released through the action of phospholipase A_2_ and serve as substrates for cyclooxygenase and lipoxygenase, giving rise to 3-series prostanoids (e.g., prostaglandin I_3_, thromboxane A_3_) and 5-series leukotrienes (e.g., leukotriene B_5_) [15]. Prostaglandin, thromboxane, and leukotriene derived from OM3 are much less potent mediators than corresponding omega-6 fatty acid derivatives, which generally have pro-inflammatory and pro-thrombotic effects. OM3 also exerts anti-inflammatory effects through promoting the resolution of inflammation [78]. OM3 is precursors of a series of lipid mediators including resolvins, protectins, and maresins [79], which are collectively named as specialized pro-resolving mediators (SPM) [80]. EPA- and DHA-derived SPMs are structurally different and interact with different receptors [81]. Administration of EPA alone without DHA in JELIS and REDUCE-IT showed a significant increase in plasma EPA but significant decrease in plasma DHA [82,83]. Thus, it may suggest that the clinical results observed with using EPA alone without DHA might be much enhanced if both EPA and DHA are administered.

All four of the studies that showed significantly slower progression of atherosclerosis were open label trials without placebo [18,19,20,26]. In this design, both investigators (physicians and staff) and participants knew the treatment assignment, which might affect the lifestyle of participants differently between the intervention and control groups. Notably, JELIS was also an open-label trial without placebo. However, the REDUCE-IT trial was a double-blind placebo-controlled trial, which showed a significant 25% relative reduction in CVD outcomes. 

Another limitation of the current meta-analysis is the fact that various technologies were used to evaluate the progression of atherosclerosis. Thus, the significantly slower progression in the synthesized SMD must be interpreted with caution. Two meta-analyses showed that reduction in coronary plaque is significantly associated with lower CHD rates [84,85]. In our meta-analysis three out of four RCTs that evaluated changes in coronary plaque showed significant reduction [18,19,20]. Although one study showed no significant difference in the progression of coronary plaque (*p* = 0.14) [17] in their per-protocol analysis, the difference approached significance (*p* = 0.07). Progression of carotid IMT has been widely used as a surrogate marker of clinical outcomes [86,87]. However, two recent meta-analyses of RCTs reported inconsistent results [88,89]. 

## 5. Conclusions

This systematic review and meta-analysis of RCTs with high-dose OM3 on atherosclerosis documents that high-dose OM3 significantly slows the progression of atherosclerosis. The results suggest that anti-atherosclerotic properties of high-dose OM3 are one potential mechanism in reducing CVD outcomes that was demonstrated in a recent RCT of high-dose OM3 on CVD outcomes. 

## Figures and Tables

**Table 1:** Characteristics of the included trials.

Author, Year, Location, Reference	Number of Participants Treatment/Control	Age (years) Treatment/Control	Characteristics of the Participants	Use of Statin	Use of Placebo	Dose and Type of OM3 (g/day)	Purity of OM3 (%)	Duration of Intervention (months)
Alfaddagh, 2017, US [17]	143/142	63 ± 8 vs. 64 ± 8	CHD or CV risk factor	Yes	No	1.86 EPA + 1.50 DHA	90%	30
Bhatia, 2016, UK [27]	51/52	49 ± 11 vs. 54 ± 9	NAFLD	No	Yes	1.368 EPA + 1.656 DHA	90%	15 to 18
Mita, 2006, Japan [26]	40/41	59 ± 11 vs. 61 ± 8	T2DM	No	No	1.8 EPA	>98%	25 ± 2
Niki, 2016, Japan [19]	48/47	68 ± 10 vs. 69 ± 11	CHD and DL	Yes	No	1.8 EPA	>98%	6
Nishio, 2014, Japan [18]	25/27	61 ± 13 vs. 64 ± 10	CHD and DL	Yes	No	1.8 EPA	>98%	9
Watanabe, 2017, Japan [20]	122/119	67 ± 10 vs. 68 ± 10	CHD	Yes	No	1.8 EPA	>98%	6 to 8

OM3: marine omega-3 fatty acids, CHD: coronary heart disease, CV: cardiovascular, NAFLD: non-alcoholic fatty liver disease, T2DM: type 2 diabetes, DL: dyslipidemia, EPA: eicosapentaenoic acid, DHA: docosahexaenoic acid.

**Table 2:** Primary outcome of atherosclerosis and the result of each included trial.

First Author, Year, Country, Reference	Imaging Techniques	Primary Outcome	Baseline Measurement Treatment vs. Control Groups	Difference in Primary Outcome between the End of Intervention and Baseline in Each of Treatment and Control Groups Treatment vs. Control Groups	Net Difference between Intervention and Control Groups	*p*-Value for Net Difference
Alfaddagh, 2017, US [17]	cCTA	Percent change in non-calcified plaque volume (%)	26.4 (14.3, 39.7) vs. 23.7 (14.3, 36.8)	1.71 ± 19.9 vs. 4.75 ± 16.44	−3.04	0.14
Bhatia, 2016, UK [27]	B-Mode ultrasound	Change in mean carotid IMT (mm)	0.649 ± 0.095 vs. 0.674 ± 0.098	0.0124 ± 0.0115 vs. 0.0157 ± 0.0138	−0.003	0.17
Mita, 2006, Japan [26]	B-mode ultrasound	Annual change in maximum carotid IMT (mm/year)	1.505 ± 0.412 vs. 1.706 ± 0.423	−0.084 ± 0.113 vs. −0.005 ± 0.108	−0.079	<0.01
Niki, 2016, Japan [19]	IVUS	Change in lipid plaque volume (mm^3^)	18.5 ± 1.3 vs. 17.8 ± 1.3	−3.5 ± 0.2 vs. 1.5 ± 1.0	−5.0	<0.01
Nishio, 2014, Japan [18]	OCT	Change in fibrous-cap thickness (um)	47.5 ± 7.4 vs. 46.5 ± 10.9	−54.8 ± 27.9 vs. −23.5 ± 11.6	−31.3	<0.01
Watanabe, 2017, Japan [20]	IVUS	Change in normalized total atheroma volume (mm^3^)	74.2 (55.9, 99.2) vs. 74.2 (57.5, 96.8)	−8.49 ± 2.37 vs. −2.90 ± 4.74	−5.59	<0.01

cCTA: coronary computed tomographic angiography, IVUS: integrated backscatter intravascular ultrasound; OCT: optical coherence tomography, IMT: intima-media thickness; SD: standard deviation; Baseline measurement is expressed as mean (SD) or median (inter-quartile range).

**Table 3:** Risk of bias for each trial.

Study	Selection Bias		Performance bias	Detection Bias	Attrition Bias	Reporting Bias	Other Bias	Total
	Random Sequence Generation	Allocation Concealment	Blinding of Participants and Personnel	Blinding of Outcome Assessment	Incomplete Outcome Data	Selective Outcome Reporting	Other Source of Bias	Low on Risk of Bias
Alfaddagh, 2017, US [17]	low	high	high	low	low	low	low	5/7
Bhatia, 2016, UK [27]	low	unclear	low	low	low	low	low	6/7
Mita, 2006, Japan [26]	unclear	unclear	high	low	low	unclear	low	3/7
Niki, 2016, Japan [19]	low	low	high	low	low	low	low	6/7
Nishio, 2014, Japan [18]	unclear	unclear	high	low	low	unclear	low	3/7
Watanabe, 2017, Japan [20]	low	high	high	unclear	low	low	low	4/7

## Supplementary Materials

The following are available online at https://www.mdpi.com/2072-6643/11/11/2599/s1: Figure S1: Effect of low-dose marine omega-3 fatty acids on atherosclerosis; Table S1: Search strategy and number of articles retrieved in PubMed; Table S2: Search strategy and number of articles retrieved in Embase; Table S3: Characteristics of the trials with low-dose OM3; Table S4: Primary outcome of atherosclerosis and the result of each trial with low-dose OM3; Table S5: Risk of bias for trails with low-dose OM3.
