# Supplementary material for: Effect of High-Dose Marine Omega-3 Fatty Acids on Atherosclerosis: A Systematic Review and Meta-Analysis of Randomized Clinical Trials
Source: Nutrients. 2019 Oct 30;11(11):2599. doi: 10.3390/nu11112599 (PMC6893789; doi:10.3390/nu11112599)
Supplement: Supplementary file 1 [file nutrients-11-02599-s001.pdf]

Supplementary Materials:

Table S1. Search strategy and number of articles retrieved in PubMed.

Table S2. Search strategy and number of articles retrieved in Embase.

Table S3. Characteristics of the trials with low-dose OM3.

Table S4. Primary outcome of atherosclerosis and the result of each trial with low-dose OM3.

Table S5. Risk of bias for trails with low-dose OM3.

Figure S1. Effect of low-dose marine omega-3 fatty acids on atherosclerosis.

Table S1. Search strategy and number of articles retrieved in PubMed.

1	fish oils[MH] OR fatty acids, omega-3[MH] OR dietary supplements[MH] OR eicosapentaenoic acid[MH] OR docosahexaenoic acids[MH] OR fatty acids[MH]	
2	fish oil[TIAB] OR fatty fish[TIAB] OR omega-3 fatty acids[TIAB] OR omega-3 fatty acid[TIAB] OR n-3 fatty acids[TIAB] OR n-3 polyunsaturated fatty acids[TIAB] OR eicosapentaenoic acid[TIAB] OR docosahexaenoic acid[TIAB] OR dietary supplementation[TIAB] OR lovaza[TIAB] OR epanova[TIAB] OR vascepa[TIAB]	
3	#1 OR #2	506,467
4	atherosclerosis[MH] OR atherosclerosis, coronary[MH] OR arteriosclerosis[MH] OR plaque, atherosclerotic[MH] OR coronary stenosis[MH] OR carotid stenosis[MH]	
5	atherosclerosis[TIAB] OR arteriosclerosis[TIAB] OR atherosclerotic plaque[TIAB] OR coronary stenosis[TIAB] OR carotid stenosis[TIAB]	
6	#4 OR #5	256,170
7	randomized controlled trial[PT] OR controlled clinical trial[PT] OR clinical trial[PT] OR clinical study[PT]	
8	controlled clinical trials as topic[MH] OR randomized controlled trial[MH] OR placebos[MH] OR double-blind method[MH]	
9	randomized[TIAB] OR placebo[TIAB] OR randomly[TIAB] OR trial[TIAB] OR blind[TIAB] OR groups[TIAB]	
10	#7 OR #8 OR #9	3,097,327
11	diagnostic imaging[MH] OR image processing, computer-assisted[MH] OR angiography[MH] OR magnetic resonance imaging[MH] OR ultrasound[MH] OR tomography[MH] OR angioscopy[MH] OR magnetic resonance spectroscopy[MH]	
12	imaging[TIAB] OR diagnostic imaging[TIAB] OR magnetic resonance*[TIAB] OR intravascular*[TIAB] OR angioscopy*[TIAB] OR tomography*[TIAB] OR angioscopy*[TIAB] OR optical*[TIAB]	
13	#11 OR #12	3,312,160
14	#3 AND #6 AND #10 #13	493

Table S2. Search strategy and number of articles retrieved in Embase.

1	'omega 3 fatty acid'/exp OR 'fish oil'/exp OR 'icosapentaenoic acid'/exp OR 'docosapentaenoic acid'/exp OR 'docosahexaenoic acid'/exp OR 'dietary supplement'/exp OR 'diet supplementation'/exp OR 'omega 3 acid ethyl ester'/exp OR 'omega 3 fatty acid ester'/exp	
2	'omega 3 fatty acid':ab,ti OR 'fish oil':ab,ti OR 'icosapentaenoic acid':ab,ti OR 'icosapentaenoic acid ethyl ester':ab,ti OR 'omega 3 acid ethyl ester':ab,ti OR 'omega 3 fatty acid ester':ab,ti OR 'docosapentaenoic acid':ab,ti OR 'docosahexaenoic acid':ab,ti OR 'fish':ab,ti OR 'dietary supplement':ab,ti OR 'diet supplementation':ab,ti	
3	#1 OR #2	306,256
4	'atherosclerosis'/exp OR 'coronary artery atherosclerosis'/exp OR 'aortic atherosclerosis'/exp OR 'brain atherosclerosis'/exp OR 'peripheral occlusive artery disease'/exp OR 'carotid atherosclerosis'/exp OR 'nephrosclerosis'/exp OR 'peripheral occlusive artery disease'/exp OR 'atherosclerotic plaque'/exp	
5	'atherosclerosis':ab,ti OR 'coronary artery atherosclerosis':ab,ti OR 'aortic atherosclerosis':ab,ti OR 'atherosclerotic plaque':ab,ti	
6	#4 OR #5	391,824
7	'diagnostic imaging equipment'/exp OR 'diagnostic imaging'/exp OR 'imaging and display'/exp OR 'nuclear magnetic resonance imaging'/exp OR 'cardiac imaging'/exp OR 'digital imaging'/exp OR 'fluorescence imaging'/exp OR 'image analysis'/exp OR 'computer assisted diagnosis'/exp OR 'echography'/exp OR 'quantitative diagnosis'/exp OR 'radiodiagnosis'/exp	
8	'diagnostic imaging equipment':ab,ti OR 'diagnostic imaging':ab,ti OR 'angiography':ab,ti OR 'arteriography':ab,ti OR 'magnetic resonance angiography':ab,ti OR 'digital subtraction angiography':ab,ti OR 'coronary angiography':ab,ti OR 'fluorescence angiography':ab,ti OR 'echography':ab,ti OR 'echocardiography':ab,ti OR 'ultrasound':ab,ti OR 'doppler glowmetry':ab,ti OR 'angioscopy':ab,ti OR 'emission tomography':ab,ti OR 'computer assisted emission tomography':ab,ti OR 'positron emission tomography':ab,ti OR 'photone emission tomography':ab,ti OR 'optical coherence tomography':ab,ti OR 'computed tomography scanner':ab,ti	
9	#7 OR #8	3,602,675
10	'clinical trial (topic)'/exp OR 'controlled clinical trial (topic)'/exp OR 'randomized controlled trial (topic)'/exp OR 'multicenter study (topic)'/exp OR 'phase 1 clinical trial (topic)'/exp OR 'phase 2 clinical trial (topic)'/exp OR 'phase 3 clinical trial (topic)'/exp OR 'phase 4 clinical trial (topic)'/exp	
11	'randomized controlled trial':ab,ti OR 'controlled clinical trial':ab,ti OR 'controlled study':ab,ti OR 'clinical trial':ab,ti OR 'placebo':ab,ti	
12	#10 OR #11	769,632
13	#3 AND #6 AND #9 AND #12	85

Table S3. Characteristics of the trials with low-dose marine omega-3 fatty acids.

**Author, year, location, reference**	**Number of participants Treatment/control**	**Age (years) Treatment / Control**	**Characteristics of the participants**	**Use of statin**	**Use of placebo**	**Dose and type of OM3 (g/day)**	**Purity of OM3 (%)**	**Duration of intervention (months)**
Ahn, 2016, South Korea [45]	38/36	60 ± 9 vs. 61 ± 1	CHD	Yes	Yes	1.395 EPA + 1.125 DHA	84%	12
Angerer, 2002, Germany [46]	112/111	57 ± 9 vs. 59 ± 8	CHD	No	Yes	1.65 (EPA + DHA)	55%	21
Baldassarre, 2006, Italy [47]	32/32	54 ± 7 vs. 54 ± 7	Hyperlipidemia without CVD	No	Yes	1.08 EPA + 0.72 DHA (5)	32%	24
Lonn, 2013, 7 countries [48]	585/599	63 vs. 63	Dysglycemia + (CVD or CV risk factors)	Yes	Yes	0.465 EPA + 0.375 DHA	84%	59
von Schacky, 1999, Germany [49]	112/111	58 ± 10 vs. 59 ± 8	CHD	No	Yes	1.062 EPA + 0.645 DHA (6)	57%	21

OM3: marine omega-3 fatty acids, CHD: coronary heart disease, CVD: cardiovascular disease, EPA: eicosapentaenoic acid, DHA: docosahexaenoic acid.

Table S4. Primary outcome of atherosclerosis and the result of each trial with low-dose marine omega-3 fatty acids.

First Author, Year, Country, reference	Imaging techniques	Primary Outcome	Baseline Measurement Treatment vs. control groups	Difference in primary outcome between the end of intervention and baseline in each of treatment and control groups Treatment vs. control groups	Net difference between treatment and control groups	*p*-value for Net difference
Ahn, 2016, South Korea [45]	IVUS	Percent change in atheroma volume index (%)	5.51 ± 5.50 vs. 7.09 ± 4.83	−12.65 ± 30.19 vs. -8.51 ± 55.5	−4.14	0.77
Angerer, 2002, Germany [46]	B-mode ultrasound	Change in mean carotid IMT (mm)	1.26 ± 0.41 vs. 1.31 ± 0.41	0.07 ± 0.13 vs. 0.05 ± 0.11	0.02	0.24
Baldassarre, 2006, Italy [47]	B-mode ultrasound	Change in mean carotid IMT (mm)	0.79 ± 0.15 vs. 0.83 ± 0.16	0.9 ± 3.29 vs. 1.6 ± 3.12	−0.7	>0.05
Lonn, 2013, 7 countries [48]	B-mode ultrasound	Change in mean carotid IMT (mm)	1.08 ± 0.33 vs. 1.10 ± 0.35	0.0254 ± 0.0348 vs. 0.0244 ± 0.0352	0.0009	>0.65
von Schacky, 1999, Germany [49]	QCA	Loss in coronary minimal luminal diameter	N/A	0.38 ± 0.8 vs. 0.45 ± 0.8	−0.07	>0.05

IVUS: intravascular ultrasound; QCA: Quantitative coronary angiography; IMT: intima-media thickness; SD: standard deviation; NS: non-significant; N/A: not available.

Table S5. Risk of bias for trails with low-dose marine omega-3 fatty acids.

Study	Selection bias		Performance bias	Detection bias	Attrition bias	Reporting bias	Other bias	Total
	Random sequence generation	Allocation concealment	Blinding of participants and personnel	Blinding of outcome assessment	Incomplete outcome data	Selective outcome reporting	Other source of bias	Low on risk of bias
Ahn, 2016, South Korea [45]	low	low	low	low	low	low	low	7/7
Angerer, 2002, Germany [46]	low	low	low	low	low	low	low	7/7
Baldassarre, 2006, Italy [47]	low	low	low	unclear	low	low	low	6/7
Lonn, 2013, 7 countries [48]	low	low	low	low	low	low	low	7/7
von Schacky, 1999, Germany [49]	low	low	low	low	high	low	low	7/7

	OM3			Control				
Study	Mean	SD	Total	Mean	SD	Total	Weight	SMD and 95%CI
Ahn 2016 [44]	−12.65	30.19	38	−8.51	55.5	36	5.1%	−0.09 [−0.55, 0.36]
Angerer 2002 [45]	0.07	0.13	87	0.05	0.11	84	11.8%	0.17 [−0.14, 0.47]
Baldassarre 2006 [46]	0.9	3.29	30	1.6	3.12	27	3.9%	−0.22 [−0.74, 0.31]
Lonn 2013 [47]	0.0254	0.0348	539	0.0244	0.0352	552	75.3%	0.03 [−0.09, 0.15]
Von 1999 [48]	0.38	0.8	29	0.45	0.8	29	4.0%	−0.09 [−0.60, 0.43]
**Total (95% CI)**			**723**			**728**	**100.0%**	**0.02 [−0.08, 0.13]**

Heterogeneity: $Tau^2 = 0.00$; $Chi^2 = 2.09$, df = 4 (P = 0.72); $I^2 = 0\%$
Test for overall effect: Z = 0.46 (P = 0.64)

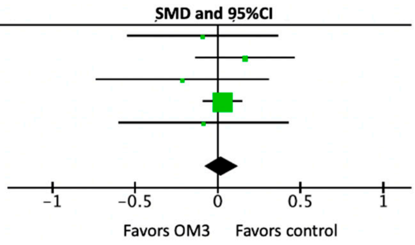

OM3: marine omega-3 fatty acids; SMD: standard mean difference; SD: standard deviation; CI: confidence interval

Figure S1. Effect of low-dose marine omega-3 fatty acids on atherosclerosis.
